# The *NF1*+/- Immune Microenvironment: Dueling Roles in Neurofibroma Development and Malignant Transformation

**DOI:** 10.3390/cancers16050994

**Published:** 2024-02-29

**Authors:** Emily E. White, Steven D. Rhodes

**Affiliations:** 1 Medical Scientist Training Program, Indiana University School of Medicine, Indianapolis, IN 46202, USA; 2 Department of Medical and Molecular Genetics, Indiana University School of Medicine, Indianapolis, IN 46202, USA; 3 Department of Pediatrics, Herman B Wells Center for Pediatric Research, Indiana University School of Medicine, Indianapolis, IN 46202, USA; 4 Division of Pediatric Hematology/Oncology/Stem Cell Transplant, Indiana University School of Medicine, Indianapolis, IN 46202, USA; 5 IU Simon Comprehensive Cancer Center, Indiana University School of Medicine, Indianapolis, IN 46202, USA

**Keywords:** neurofibromatosis type 1 (NF1), plexiform neurofibroma, atypical neurofibroma, malignant peripheral nerve sheath tumor, Schwann cells, immune microenvironment, mast cells, macrophages, T cells, immunotherapy, biomarkers

## Abstract

**Simple Summary:**

In this review, we explore how interactions between tumorigenic Schwann cells and infiltrating immune cells shape the development and malignant transformation of peripheral nerve sheath tumors in neurofibromatosis type 1. We summarize the current state of the field and address key knowledge gaps surrounding the impact of neurofibromin haploinsufficiency on immune cell function, as well as the impact of Schwann cell lineage states on immune cell recruitment and activation within the tumor microenvironment. Furthermore, we discuss emerging evidence suggesting a dueling role of the immune system in promoting benign tumor initiation while potentially restraining malignant outgrowth. Finally, we highlight the potential implications of these findings and suggest future directions for research relevant to the diagnosis, risk-assessment, and treatment of peripheral nerve sheath tumors, utilizing immunomodulatory therapeutics.

**Abstract:**

Neurofibromatosis type 1 (NF1) is a common genetic disorder resulting in the development of both benign and malignant tumors of the peripheral nervous system. NF1 is caused by germline pathogenic variants or deletions of the *NF1* tumor suppressor gene, which encodes the protein neurofibromin that functions as negative regulator of p21 RAS. Loss of *NF1* heterozygosity in Schwann cells (SCs), the cells of origin for these nerve sheath-derived tumors, leads to the formation of plexiform neurofibromas (PNF)—benign yet complex neoplasms involving multiple nerve fascicles and comprised of a myriad of infiltrating stromal and immune cells. PNF development and progression are shaped by dynamic interactions between SCs and immune cells, including mast cells, macrophages, and T cells. In this review, we explore the current state of the field and critical knowledge gaps regarding the role of *NF1(Nf1)* haploinsufficiency on immune cell function, as well as the putative impact of Schwann cell lineage states on immune cell recruitment and function within the tumor field. Furthermore, we review emerging evidence suggesting a dueling role of *Nf1+/-* immune cells along the neurofibroma to MPNST continuum, on one hand propitiating PNF initiation, while on the other, potentially impeding the malignant transformation of plexiform and atypical neurofibroma precursor lesions. Finally, we underscore the potential implications of these discoveries and advocate for further research directed at illuminating the contributions of various immune cells subsets in discrete stages of tumor initiation, progression, and malignant transformation to facilitate the discovery and translation of innovative diagnostic and therapeutic approaches to transform risk-adapted care.

## 1. Introduction

Neurofibromatosis type 1 (NF1) is an autosomal-dominant cancer predisposition syndrome characterized by the propensity to develop tumors throughout the central and peripheral nervous system, affecting approximately 1 in 3000 people worldwide [1]. It is caused by pathogenic variants in the *NF1* tumor suppressor gene on chromosome 17, which encodes the protein neurofibromin [2,3,4,5]. Neurofibromin acts as a GTPase-activating protein (GAP) for p21 RAS, accelerating the hydrolysis of active GTP-bound RAS to its inactive GDP-bound form to regulate cell proliferation and survival [2,3,4,5]. Loss of neurofibromin results in hyperactivation of RAS-dependent signaling and leads to the development of a myriad of disease-related manifestations.

One of the most common and debilitating manifestations of NF1 is the development of plexiform neurofibromas (PNF), which are benign tumors arising from Schwann cells, comprising the peripheral nerve sheath. PNF can result in a range of morbidities, including pain, disfigurement, and functional impairment [6]. In the majority of affected individuals, PNF grow slowly or stop growing entirely once patients reach adulthood [7]. However, a subset of persons with NF1 develop atypical neurofibromas (ANF), which can undergo transformation to a lethal form of sarcoma, called malignant peripheral nerve sheath tumor (MPNST). The lifetime incidence of MPNST in individuals with NF1 is estimated to be between 8–16% [8,9]. MPNST is the leading cause of premature death in NF1 patients, with a 5-year survival rate of 20–50% [8,9]. Surgical excision with wide margins represents the only prospect for cure for these highly aggressive sarcomas, which are resistant to conventional chemotherapy and radiation. Hence, understanding the cellular and molecular mechanisms that drive progression of tumors along the neurofibroma to MPNST continuum is critical to developing biomarkers that identify tumors at high risk of undergoing malignant transformation and to expedite the clinical translation of new and effective therapies to improve treatment outcomes in patients.

Genetic alterations associated with neurofibroma genesis and malignant transformation have been extensively studied, and several key driver events have been implicated. Persons with NF1 are born with a heritable or de novo germline pathogenic variant or microdeletion of one *NF1* allele. Subsequently, loss of heterozygosity (LOH) of *NF1* is acquired either in utero or postnatally in tumorigenic Schwann cells (SCs), resulting in the development of PNF [10,11,12,13], which often manifests in early childhood. Contrastingly, atypical neurofibromas often arise beginning in adolescence as distinct nodular lesions (DNLs) that emerge from within an existing PNF. These DNLs often exhibit increased FDG-PET avidity, and biopsy or surgical resection reveals the presence of characteristic histopathologic features, including nuclear atypia, hypercellularity, loss of neurofibroma architecture, and a mitotic rate >1/50 and <3/10 HPF [14,15]. Lesions with isolated atypia are termed neurofibroma with atypia, whereas lesions harboring two or more of these features are termed atypical neurofibromatous neoplasms of uncertain biologic potential (ANNUBP). Microarray-based comparative genome hybridization (aCGH) and, more recently, whole exome sequencing have shown that these lesions frequently harbor copy number loss of the *CDKN2A/B* tumor suppressor locus [16,17]. Preclinical studies in genetically engineered mouse models (GEMMs) have confirmed *Cdkn2a* loss to be a key inciting event in ANNUBP development and malignant transformation [18,19]. Additionally, pathogenic variants in PRC2 complex-associated genes, including *EED* and *SUZ12*, have been frequently identified in MPNST [20,21,22,23] but are not characteristically present in ANNUBP or PNF [16,17].

While these genetic alterations represent key driver events responsible for promoting peripheral nerve sheath tumor (PNST) progression, the incidence of MPNST in individuals who develop atypical neurofibroma is only ~50% by 51 years of age and with variable latency [24]. Notably, in this cohort of 63 patients, 17 developed MPNST in a location distinct from their atypical neurofibroma [24]. Moreover, NF1 patients often present clinically with multiple DNLs, yet not all DNLs progress uniformly to MPNST. Similarly, in genetically engineered mice harboring heterozygous *Cdkn2a* loss, only about 50% of these mice develop MPNST [18,19]. Therefore, given the heterogeneous behavior of these precursor lesions in both patients and mice, it is plausible that additional factors, including epigenetic changes within the tumorigenic Schwann cells themselves or within the tumor microenvironment (TME), may also play a critical role in modulating the growth and malignant potential of PNF and ANNUBP.

The TME is recognized to play a key role in neurofibroma pathogenesis. *NF1* haploinsufficient (*Nf1+/-*) immune and stromal cells exhibit multiple RAS-dependent gains in function in response to various cytokines, which are present at increased levels within the serum of NF1 patients and GEMMs [25,26]. Here, we review the current state of the field, discuss key knowledge gaps pertaining to the impact of *NF1(Nf1)* haploinsufficiency on immune cell function, and how heterotypic Schwann cell–immune cell interactions may serve to critically govern PNF pathogenesis and malignant transformation. Ultimately, a more detailed understanding of the role of the immune microenvironment in shaping neurofibroma growth and progression could lead to new opportunities for PNST treatment in the realm of immunomodulatory therapeutics.

## 2. Biallelic Loss of *NF1* in Schwann Cell Precursors Is Fundamental to PNF Development

PNF are benign tumors involving multiple nerve fascicles that can occur in up to 50% of persons with NF1 [27]. These complex tumors are composed of multiple cell types, including Schwann cells, fibroblasts, mast cells, macrophages, lymphocytes, neurons, endothelial cells, and pericytes [28]. GEMMs have played a critical role in delineating Schwann cells (SCs) as the cell of origin for PNF. *Nf1+/-* mice do not develop PNF, suggesting that *Nf1* LOH in at least a subset of cells is required for the PNF development [29]. *Nf1-/-* mice die of cardiac failure at embryonic day 13.5 and are thus not useful for studying PNF development or identifying the specific cell types responsible for tumor initiation [30]. Ultimately, the development of conditional knockout mice harboring a *Cre* transgene driven by the *Krox20* promoter showed that biallelic loss of *Nf1* in the SC lineage leads to PNF development [31]. Notably, PNF development in this model additionally required *Nf1* heterozygosity (*Nf1+/-*) in non-neoplastic cells, suggesting a pivotal role of the *Nf1* haploinsufficient tissue field in tumor initiation and progression. Subsequently, biallelic ablation of *Nf1* using a variety of neural crest-specific promoters to drive Cre-mediated recombination in SCs and Schwann cell precursors (SCPs) has been shown to recapitulate PNF development [31,32,33,34,35,36,37]. Complementary approaches, deleting *Nf1* exogenously in skin-derived precursor cells and dorsal root ganglion nerve-root neurosphere cells, followed by reintroduction to the sciatic nerve, achieved similar results [38]. Recently, PNF formation was also achieved using CRISPR-Cas9 knockout (KO) of *NF1* in human-induced pluripotent stem cell (iPSC)-derived SCPs, implanted orthotopically into the sciatic nerve of immunocompromised recipient mice [39]. Collectively, these studies implicate SCs and their precursors as the cells responsible for PNF initiation.

PNF usually develop in early childhood and can grow throughout adolescence [40]. *Nf1-/-* SC proliferation is driven largely through hyperactivation of RAS/MAPK-dependent signaling [41]. RAS signals to a myriad of downstream effectors, including rapidly accelerated fibrosarcoma kinase (RAF) [42]. RAF is a serine/threonine kinase that phosphorylates MEK on serine residues. Subsequently, MEK phosphorylates ERK, a MAPK effector of pathways involved in cell growth and proliferation [43,44].

Substantial preclinical and clinical evidence supports the role of deregulated RAS/MAPK signaling in neurofibroma formation and growth. ERK-dependent target genes are overexpressed in both murine and human PNF, and both MEK and ERK inhibitors have been shown to block PNF growth in vivo [45,46]. Furthermore, multiple phase I and II clinical trials have demonstrated the efficacy of MEK inhibitors (selumetinib, mirdametinib, trametinib, and binimetinib) in the treatment of PNF, resulting in tumor shrinkage, functional benefits, and improvement in patient-reported outcome measures, including pain and quality of life [47,48,49,50,51]. In 2020, the United States Food and Drug Administration (FDA) approved selumetinib for the treatment of symptomatic, inoperable PNF in children 2 years of age and older [52].

Another signaling axis implicated in PNF development is the PI3K/AKT/mTOR pathway [53]. In NF1, this pathway is involved in cell cycle progression and survival of tumorigenic SCs. Importantly, co-inhibition of the MAPK and PI3K/AKT/mTOR pathways using a MEK inhibitor and everolimus, an mTOR inhibitor, showed a reduction in tumor burden in two different MPNST-forming GEMMs [54]. A phase II clinical trial investigating the use of another mTOR inhibitor, sirolimus, in patients with progressive PNF showed a modest improvement in time to progression [55]. SARC031, a phase II clinical trial of the MEK inhibitor selumetinib in combination with sirolimus in MPNST, has completed enrollment. However, the final results have not been published yet at the time of writing this review. Collectively, these data suggest that multiple RAS-dependent effector pathways cooperate to drive aberrant cellular proliferation and survival in NF1-associated PNST.

## 3. Neurofibromin Governs SC–Immune Cell Interactions

In addition to intracellular signaling pathways, loss of neurofibromin in SCs also critically alters paracrine-signaling networks governing the interactions between SCs and immune cells within the tumor microenvironment. *Nf1-/-* SCs secrete a plethora of inflammatory cytokines and paracrine factors, including SCF (c-kit ligand), CSF1, VEGF, and RANTES, which promote mast cell and macrophage recruitment to the PNF microenvironment [56,57]. As immune cells enter the tissue field, they induce further alterations in the local milieu, potentiating the growth of neoplastic SCs [58].

Bulk and single-cell RNA sequencing have delineated complex chemokine and paracrine signaling networks, orchestrating immune cell recruitment to the PNF microenvironment. *Nf1-/-* SCs overexpress pleiotropin and midkine, homologous ligands that bind to receptor tyrosine kinases, serve as potent mitogens for neurofibroma derived cells [59], and are known to increase in response to inflammation and NF-κB-dependent signaling [60]. Evidence suggests that midkine may also stimulate inflammatory responses and promote the recruitment of multiple immune cell types, including T cells, B cells, and macrophages, to the tumor microenvironment [61,62,63,64]. PNF arising in *Dhh-Cre;Nf1^flox/flox^* mice exhibit increased immune cell infiltration and dynamic changes in immune cell proportions over time [60]. Specifically, macrophage subpopulations were found to comprise a greater proportion of total immune cell infiltrates in PNF from 7-month-old *Dhh-Cre;Nf1^flox/flox^* mice relative to both age-matched WT littermates and 2-month-old *Dhh-Cre;Nf1^flox/flox^* pre-tumor controls. Contrastingly, myeloid-derived suppressor cells were reduced in PNF from older *Dhh-Cre;Nf1^flox/flox^* mice. Genes encoding transcription factors that regulate key inflammatory programs, including Jun, NF-κB, and Yy1, were differentially expressed in PNF compared to controls. Notably, multiple NF-κB effectors (*Nfkbia*, *Nfkb*, *Rela*) were upregulated in PNF SCs associated with enhanced STAT3 pathway activation. Human PNF demonstrated similar features, with an even greater degree of immune cell infiltration in comparison to that observed in murine PNF [60].

In summary, *Nf1-/-* SCs exhibit enhanced pro-inflammatory transcriptional programs that mediate immune cell recruitment to the PNF microenvironment though distinct receptor-ligand interactions. Single cell transcriptomic analysis has further revealed diverse SC populations in PNF, each harboring distinct expression profiles and predicted cell–cell communication networks [60]. Collectively, these findings suggest that discrete SC populations may interact uniquely with various immune cell subsets within the tumor microenvironment.

## 4. SC Lineage Fates Influence Neurofibroma Genesis and Immune Cell Cross Talk

**Figure 1:**
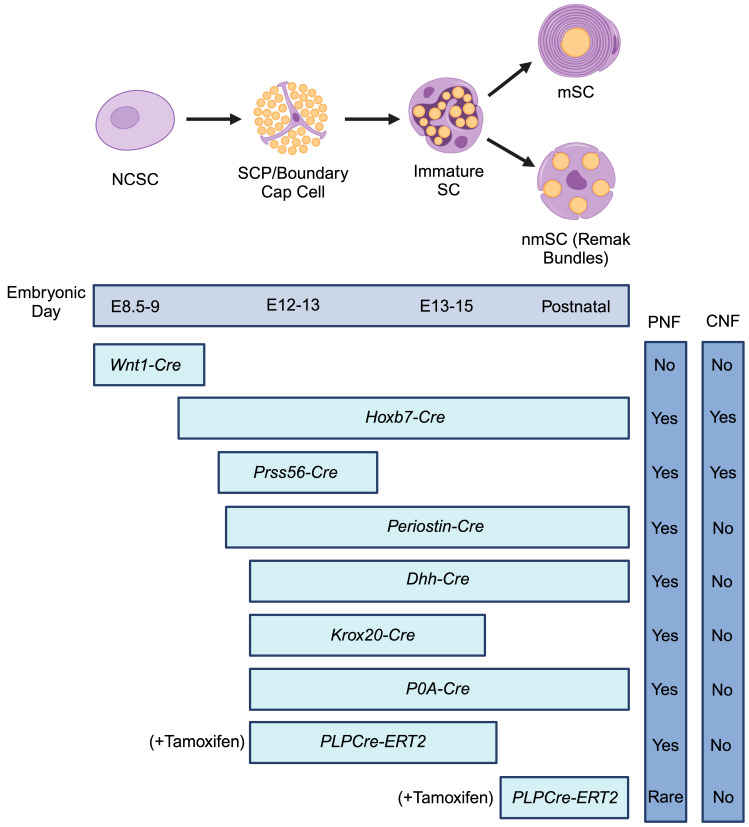
Schematic illustrating stages of Schwann cell differentiation in relation to neurofibroma genesis. The top bar indicates the embryonic day at which the Schwann cells and their precursor first appear. The next series of bars indicates the stages at which various promoters drive Cre-mediated recombination of *Nf1* in transgenic mouse models. The vertical bars denote whether the mouse model results in the formation of plexiform neurofibroma (PNF), cutaneous neurofibroma (CNF), or both. Schema adapted from Carroll et al. [65] and Le et al. [36]. Created with BioRender.com.

PNF derived SCs exist along a continuum from precursor cells to mature myelinating and non-myelinating SCs [65] (Figure 1). At embryonic day 12–13, neural crest cells give rise to Schwann cell precursors (SCPs), which closely resemble neural crest stem cells (NCSCs), but also exhibit features characteristic of immature SCs [66,67]. Subsequently, SCPs further differentiate into immature SCs at embryonic day 13–15 [68]. Postnatally, immature SCs bifurcate along either myelinating or non-myelinating trajectories, depending on the diameter of contacting axons [68]. Immature SCs with a small SC-to-axon ratio differentiate into mature non-myelinating SCs (nmSCs), while immature SCs with a large SC-to-axon ratio become mature myelinating SCs (mSCs) [69].

*Nf1* loss in mature nmSCs, also known as Remak bundles, triggers abnormal proliferation that results in PNF formation [33]. Moreover, *Nf1* loss also alters SC–axon interactions in both nmSC and mSCs, contributing to tumorigenesis [70]. This deregulated SC–axonal contact is mediated by downregulation of semaphorin 4F (Sema4F), which functions physiologically to guide SC–axonal contact [70]. Reduced Sema4F expression impairs the formation and maintenance of SC–axon interactions, leading to increased neoplastic SC proliferation. Studies by Joseph and Zheng et al. suggest that nmSCs could represent the initiating cells for PNF [33,37]. While these studies implicate differentiated SCs as key contributors to PNF pathogenesis, other evidence indicates that earlier SC lineage states may also play a critical role in PNF initiation.

Studies using transgenic mice with tamoxifen-inducible Cre recombinase under the control of the myelin proteolipid protein promoter region (*PLPCre-ERT2*) have shown that introducing *Nf1* loss at distinct stages of SC development has differing effects on PNF initiation and growth [36]. In this model, *Nf1* loss in SCPs and immature SCs induces a robust PNF formation. Similarly, various promoters, including *Krox20* [31], *Periostin* [35], *Dhh* [32], and *P0A* [33], which induce Cre recombinase expression in SCPs, also result in a strong PNF phenotype. Contrastingly, when tamoxifen induction is delayed in the *PLPCre-ERT2* model, *Nf1* ablation in mature SCs of adult mice produces PNF at a much lower frequency [36]. Notably, *Nf1*-deleted NCSCs from *Wnt1-Cre* transgenic mice exhibit a transient increase in self-renewal but fail to form PNF upon transplantation [37]. Collectively, these studies provide evidence of a temporal window in which loss of *Nf1* in the SC lineage can induce PNF [36] (Figure 1).

Recently, novel transgenic models have been developed that provide further insight into neurofibroma cells of origin and recapitulate the genesis of an expanded spectrum of NF1-associated tumors, including both PNF and cutaneous neurofibromas (CNF). Whereas PNF arise in only 30–50% of persons with NF1, CNF originate from SCs in the dermis and occur in nearly all (>95%) individuals with NF1 [71]. Historically, mouse models of PNF formation have failed to produce CNF, suggesting either distinct cells of origin for the two types of neurofibroma or a yet unidentified early SCP that can give rise to both PNF and CNF [72]. Recent work has addressed this question by showing that CNF and PNF can originate from a common cell type: either a primitive SCP or a boundary cap cell [73,74]. Chen et al. utilized a *Hoxb7-Cre* to ablate *Nf1* and demonstrated that skin-derived neural progenitors (SKPs), neural crest derived stem cells that reside in the dermis, represent a common neurofibroma cell of origin [73]. Concurrently, Radomska et al. used a *Prss56-Cre* driver to delete *Nf1*, which identified boundary cap cells as a common cell of origin for PNF and CNF [74]. Boundary cap cells represent another type of neural crest-derived cell that are found in clusters at the neural tube surface, forming a barrier between the central and peripheral nervous systems [75]. During embryonic development, these boundary cap cells differentiate into SCPs that line the dorsal and ventral roots [67]. Collectively, these studies suggest that a common early-stage SCP can give rise to both PNF and CNF, depending on the location and timing of *Nf1* deletion [72] (Figure 1).

The identification of discrete SC lineage fates responsible for neurofibroma initiation raises the question of how SC lineage commitment and maturation may influence inflammatory and paracrine signals mediating SC–immune cell cross talk. Following a transient burst of increased proliferation, *Nf1* loss induces a state of senescence growth arrest in SCs [18]. The senescence-associated secretory phenotype (SASP) is a hallmark of senescent cells that promotes tumor progression and inflammation in a variety of disease states [76]. The SASP is induced via NF-κB, p53, C/EBP-dependent transcriptional programs that promote the secretion of pro-inflammatory cytokines and chemokines, including IL-6, IL-7, IL-8, and IFNs [77]. In melanoma, IFN-γ and TNF-α promote the expression of SASP-related genes, including interleukins (*IL1B* and *IL8*) and chemokines (*CXCL10* and *CXCL11*) [78]. In other cancers, oncogenic RAS and functional loss of the p53 tumor suppressor protein enhance SASP-dependent paracrine signaling [77]. It is conceivable that senescent *Nf1-/-* SCs in PNF may drive pro-inflammatory programs that promote immune cell recruitment and activation within the tumor microenvironment [58]; however, the role of senescent SCs in PNF development and progression has yet to be definitively evaluated.

Studies in the field of nerve injury provide additional insights regarding the potential influence of SC lineage states on immune cell cross talk in NF1-associated PNST. In the peripheral nervous system, nerve injury induces myelinating and non-myelinating SCs to de-differentiate to an immature state in order to facilitate repair [79,80]. These repair SCs, also known as Bungner SCs for the axonal regeneration bands they form, promote axonal elongation in injured nerves [81]. To achieve axonal repair, Bungner SCs secrete pro-inflammatory cytokines that recruit macrophages to the injury site for debris clearance [82]. However, the pro-inflammatory effects of repair SCs must be counterbalanced by neurotrophic factors, such as pituitary adenylyl cyclase-activating peptide (PACAP), which temper the pro-inflammatory role of Bungner SCs by upregulating anti-inflammatory cytokines and downregulating pro-inflammatory cytokines [83]. PACAP knockout mice exhibit impaired nerve regeneration and increased expression of IFN-γ, TNF-α, and IL-6 [84]. Interestingly, previous studies have linked SC de-differentiation during nerve injury to PNF tumorigenesis. Studies using a tamoxifen-inducible Cre recombinase, driven by the P0 promoter to disrupt *Nf1* in mSCs, showed that *Nf1-/-* repair mSCs promote neurofibroma formation at the injured nerve [85]. Thus, the inflammatory programs of de-differentiated SCPs modulated by counterregulatory neurotrophic factors may play a key role in governing PNF development.

## 5. Infiltrating Immune Cells within the TME Influence Neurofibroma Initiation

**Figure 2:**
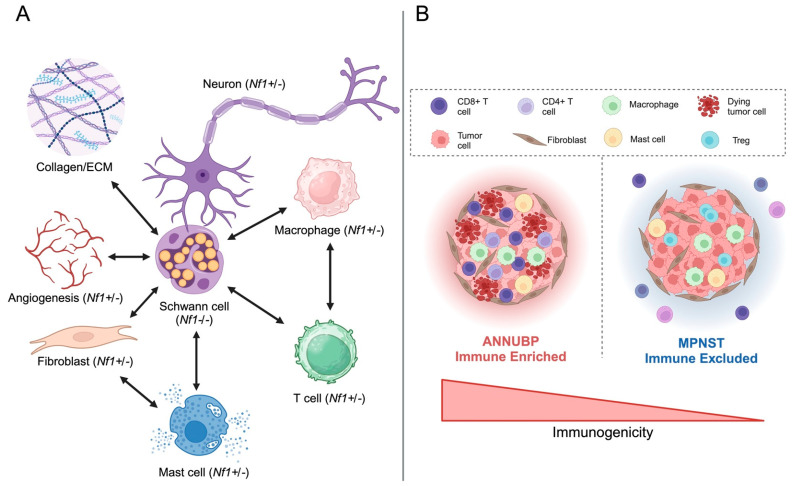
Complex cellular interactions between SCs and the tumor microenvironment shape neurofibroma development and malignant transformation. (**A**) *Nf1*-deficient SCs, the cells of origin for PNF, communicate via paracrine signaling and direct cell–cell contact with multiple *Nf1+/-* cell types within the tumor field, including neurons, macrophages, T cells, mast cells, fibroblasts, and endothelial cells, influencing neurofibroma pathogenesis. Adapted from Rhodes et al. [89]. (**B**) Malignant transformation of plexiform and atypical neurofibroma (ANNUBP) precursor lesions is associated with exclusion and/or exhaustion of infiltrating T cells, predominance of M2, pro-tumorigenic macrophages, and a decline in antigen presentation. Created with Biorender.com.

The immune microenvironment can exhibit diverse and context-dependent effects on tumor growth. NF1 provides a unique framework to explore these complexities, as systemic *NF1* heterozygosity (*NF1*+/-) fundamentally alters the host immune system. Individuals with NF1 are born with a germline pathogenic variant in one *NF1* allele, which results in haploinsufficiency in all cell types, including immune cells. Work by multiple laboratories has demonstrated that the *Nf1+/-* microenvironment accelerates or, in some cases, is even required for the genesis of multiple benign NF1-associated tumors, including PNF [31,86,87], optic nerve glioma [88], and papilloma formation [87], in response to carcinogenic insult. The *Nf1+/-* immune cells that contribute to the initiation, progression, and malignant transformation of NF1-associated tumors will be the focus of the remainder of this review, which include, but are not limited to, mast cells, macrophages, and T cells (Figure 2A).

Mast cells are one of the principal immune cell types identified in the neurofibroma microenvironment, and their role in NF1-associated tumorigenesis has been extensively studied. *Nf1-/-* SCs hypersecrete stem cell factor (SCF), the ligand for c-kit, which is critical for mast cell development and survival [90]. *Nf1* haploinsufficient mast cells exhibit enhanced migration, proliferation, survival, and degranulation in response to SCF in a Rac2/PI3K-dependent fashion [91,92,93]. Among the effector proteins hypersecreted by *Nf1+/-* mast cells is TGF-beta (TGFβ), which promotes enhanced proliferation and collagen production by *Nf1+/-* fibroblasts [93], a hallmark feature of both CNF and PNF. Additionally, mast cells secrete various pro-angiogenic growth factors in excess, including vascular endothelial growth factor (VEGF) and metalloproteinases (MMPs), which could be contributing to PNF tumorigenesis [94,95,96].

Studies in *Nf1+/-* mast cells were among the first to demonstrate the critical role of *Nf1* haploinsufficiency in mediating gains of function in non-neoplastic cells [91,97], yet genetic and pharmacologic approaches to target mast cells in PNF development have yielded differing results. In *Nf1 ^flox/^-;Krox20Cre* mice, which spontaneously develop PNF, the genetic ablation of c-kit in hematopoietic cells strikingly prevented PNF formation [86]. Similarly, pharmacologic inhibition of c-kit with imatinib attenuated PNF growth in *Nf1^flox/^-; Krox20Cre* [86] and *Nf1^flox/flox^;PostnCre* mice [98] and resulted in volumetric tumor reduction in a subset of PNF in a subsequent phase 1–2 clinical trial [99]. Additionally, the use of ketotifen, a mast cell-stabilizing agent and a first-generation antihistamine used to treat asthma and allergic disorders, reduced symptoms of pain and itch in patients with CNF [100]. In preclinical PNF models, however, ketotifen did not reduce mast cell numbers or degranulation and failed to prevent PNF formation or alter the growth of established PNF [35]. Furthermore, the genetic ablation of *Scf* in SCs was shown to decrease mast cell infiltration, but ultimately did not alter PNF formation of growth [101]. Thus, further investigation is needed to delineate the role of mast cells more clearly in the neurofibroma microenvironment.

Macrophages are among the most abundant immune cells in PNF [101]. Macrophages have been implicated as a source of excess TGFβ production within the neurofibroma microenvironment, thereby contributing to PNF growth by promoting excess collagen and extracellular matrix (ECM) deposition [102]. However, the role of macrophages in PNF initiation is complex. Tumor-associated macrophages can differentiate along either M1 (pro-inflammatory/anti-tumorigenic) or M2 (anti-inflammatory/pro-tumorigenic) trajectories [103]. Studies suggest that the PNF microenvironment is comprised primarily of M1, pro-inflammatory macrophages [101]. Intriguingly, treatment of *Dhh-Cre;Nf1^flox/flox^* mice with PLX3397, an inhibitor of the CSF1 receptor (required for macrophage recruitment, proliferation and survival) [104], administered either pre- or post-PNF initiation, yielded differing results, suggesting a temporal role of macrophage infiltration in neurofibroma growth [57]. Treatment with PLX3397 prior to PNF formation did not prevent tumor initiation and unexpectedly accelerated PNF development [57]. Contrastingly, administration of PLX3397 in an established PNF resulted in enhanced cell death and volumetric tumor regression [57]. Collectively, these findings support a temporal role of macrophages in PNF development, whereby macrophages may initially protect against PNF development, but subsequently exhibit tumor-promoting effects following PNF initiation. As the role of macrophages in neurofibroma pathogenies continues to be explored, further investigation is needed to delineate the temporal roles of specific macrophage subsets in PNF initiation and progression.

While the role of mast cells and macrophages in PNF development have been widely investigated, T cells represent a relatively understudied immune cell type within the neurofibroma microenvironment. T cells comprise approximately 4% of the total immune cell population in PNF [60]. Quantification of circulating lymphocyte populations in the peripheral blood of NF1 patients using flow cytometry revealed increased effector CD8+/CD27- and activated CD8+/CD57+ T-cell populations in subjects with low PNF and CNF tumor burden compared to patients with high tumor burden [105]. Intriguingly, in preclinical models, CXCR3-expressing leukocytes, including T cells and dendritic cells, appear to be required for PNF formation in mice [106]. *Nf1+/-* T cells are hyperproliferative in response to anti-CD3 stimulation in comparison to wild-type controls, and *Nf1+/-* mice exhibit enhanced populations of activated CD8+ T lymphocytes in response to the administration of T cell antigens in vivo [87].

Further insights into the putative role of T cells in NF1 tumor initiation can be gleaned from studies in NF1-associated low grade gliomas (LGGs). LGGs, including optic pathway gliomas (OPGs), are the most prevalent central nervous system tumors in persons with NF1 and share overlapping genetic and microenvironmental features that closely parallel PNF pathogenesis [107]. Studies have shown that midkine-dependent activation of CD8+ T cells is critical for LGG development in mice [62]. Intriguingly, athymic mice are unable to support engraftment of optic LGG stem cells secondary to impaired microglial function. Notably, T cell reconstitution restores CCR2 and CCL5 expression in microglia, allowing for LGG growth [108]. Furthermore, molecular profiling of human NF1-associated LGG revealed a subgroup of tumors with enrichments in immune-related transcripts, increased T cell infiltration, and abundant tumor neoantigens [109]. In summation, recent studies exploring the contribution of T cells to early PNF and LGG initiation suggest a critical role for T cells in the NF1 tumor microenvironment, and future research is needed to further characterize the phenotype and function of T cell subsets across the PNST continuum.

## 6. Immune Cell–SC Interactions Influence Malignant Transformation of Neurofibroma

Somatic *NF1* pathogenic variants are found in a variety of malignancies, including desmoplastic melanoma, lung cancer, and ovarian carcinoma [110], yet these sporadic cancers are not commonly associated with the NF1 tumor predisposition syndrome. The apparent contradiction of these epidemiological findings with the conventional paradigm of a strictly pro-tumorigenic role for germline *NF1* pathogenic variants requires a novel framework to account for both the pro-tumorigenic and protective effects of *NF1* mutations within the tissue field [87].

Conflicting effects of the *Nf1* heterozygous (*Nf1+/-*) immune environment on MPNST development have been observed in various models. Orthotopic injection of adenovirus expressing Cre recombinase (Ad-Cre) into the sciatic nerves of *Nf1^f/^-;Ink4a/Arf^f/f^* mice with a *Nf1+/-* microenvironment caused MPNST to develop more rapidly resulted and with enhanced CD45+ immune cell infiltrates, in comparison to *Nf1^f/f^;Ink4a/Arf^f/f^* littermates with a *Nf1*-proficient (WT) background [111]. Adoptive transfer of bone marrow from *Nf1^f/^-;Ink4a/Arf^f/f^* and *Nf1^f/f^;Ink4a/Arf^f/f^* donor mice into *Nf1^f/f^;Ink4a/Arf^f/f^* recipients confirmed these findings, suggesting that the *Nf1* haploinsufficient microenvironment accelerates MPNST development in the context of Ad-Cre injection [111].

Contrastingly, *Nf1* haploinsufficiency appears to inhibit malignant transformation in other models of NF1-associated tumorigenesis [87]. In a two-step 7,12-dimethylbenz[a]anthracene/12-*O*-tetradecanoylphorbol-13-acetate (DMBA/TPA) skin carcinogenesis model, the *Nf1+/-* microenvironment exhibited dueling roles by accelerating benign papilloma formation, while antagonizing malignant transformation to squamous cell carcinoma [87]. Moreover, in *Nf1*-floxed *PLPCre-ERT2* mice that recapitulate the spontaneous progression of PNF to MPNST, mice with a heterozygous *Nf1+/-* background (*PLPCre-ERT2*;*Nf1^f/^-*) develop PNF more rapidly than mice with a *Nf1* wild-type background (*PLPCre-ERT2;Nf1^f/f^*). Yet strikingly, despite enhanced neurofibroma genesis, 0 out of 104 *PLPCre-ERT2;Nf1^f/^-* mice developed MPNST. Conversely, 10% of *PLPCre-ERT2*;*Nf1^f/f^* mice (11 out of 112 animals) ultimately succumbed to malignancy, suggesting that the *Nf1+/-* microenvironment accelerates formation of benign tumors but restrains malignant transformation [87]. The authors further showed that anti-CD3 stimulation induces hyperproliferation of *Nf1+/-* T cells relative to WT controls. Additionally, populations of activated CD8+ T cells were increased in *Nf1+/-* mice following exposure to T cell antigens, suggesting that enhanced T cell mediated immune surveillance in the setting of *Nf1* haploinsufficiency could contribute to preventing malignant outgrowth [87].

The functional role of various T cell subsets across the neurofibroma to MPNST continuum in NF1 has yet to be fully explored. Evaluation of immune cell infiltrates in a tissue microarray comprised of 141 tissue specimens, including both NF1 and non-NF1-associated MPNSTs, neurofibromas, schwannomas, and normal nerves from 86 patients, showed that CD8+ infiltrates were significantly increased in benign PNST and MPNST vs. normal nerve [112]. PD-L1, a marker of immune exhaustion, was significantly enriched in MPNST as well [112]. Haworth and colleagues observed a correlation between CD8+/CD4+ T-cell infiltration and MPNST grade, whereby high grade MPNSTs exhibited reduced CD4+ and CD8+ T cell infiltrates in comparison to low grade MPNSTs from the same patient [113]. Recently, studies have shown that intra-tumoral T cell populations vary along the neurofibroma to MPNST continuum, based on tumor histology. Atypical neurofibroma (ANF)/ANNUBP exhibit increased numbers of CD3+ lymphocytes [114], including both CD4+ and CD8+ T cell subsets, in comparison to PNF and MPNST [115]. T-cell infiltration in MPNST is heterogenous. While some MPNST demonstrate enhanced T cell infiltrates, others appear to be largely devoid of T cells, with a predominance of FOXP3+ cells, a marker of Tregs [113,115] (Figure 2B). Transcriptomic analysis of T cell signatures revealed that a majority of human MPNST (62 out of 73 samples profiled) exhibited an immunologically “cold” phenotype, characterized by reduced cytotoxic T cell infiltrates and increased tumor immune dysfunction and exclusion (TIDE) scores [116].

A preclinical model of MPNST development demonstrated that combined cyclin-dependent kinase 4/6 (CDK4/6) and MEK inhibition sensitized MPNST to anti-PD-L1 immune checkpoint blockade (ICB) [117]. Case studies have reported deep and/or or complete responses to pembrolizumab, a PD-1 receptor inhibitor, in the treatment of PD-L1 positive relapsed/refractory MPNST [118,119,120], and several clinical trials of ICB in MPNST are currently underway. In a phase I study (NCT04465643), neoadjuvant nivolumab (a PD-1 inhibitor) and ipilimumab (a CTLA-4 inhibitor) are being administered in a window trial to patients with newly diagnosed ANNUBP and MPNST for which surgical resection is indicated. This study will establish the safety and feasibility of nivolumab and ipilimumab combination therapy, the objective response rate after receiving two doses of nivolumab and ipilimumab prior to surgery, and will evaluate biomarkers of pharmacodynamic activity, including the quantification of T cells and other immune cell subsets in the tumor and peripheral blood. Additionally, the safety and efficacy of alrizomaldin (APG-115), an MDM2 inhibitor, in combination with pembrolizumab, is being assessed for the treatment of metastatic melanomas and advanced solid tumors, including MPNST, in an ongoing phase Ib/II study (NCT03611868). Interim results revealed stable disease for > 4 cycles in 40% of the MPNST cohort (4 out of 10 patients evaluable for efficacy) [121]. These preliminary data demonstrate the potential promise of immunotherapy in at least a subset of MPNST. However, more robust biomarkers are needed to identify which patients are most likely to derive clinical benefit.

The ability of neoplastic SCs to provoke an immune response is another critical factor that may influence T-cell infiltration into the PNST microenvironment. Immunohistochemical staining of HLA-A/B/C and β2-microglobulin (B2M)—major histocompatibility complex (MHC) genes involved in antigen presentation, T cell recruitment and activation—revealed that benign neurofibromas with nodular histology and MPNST exhibited a higher average expression of HLA-A/B/C compared to diffuse and plexiform neurofibromas [113]. Notably, nodular neurofibromas exhibited the highest B2M scores of all tumor types assayed. HLA-A/B/C and B2M-staining scores correlated with CD4+, CD8+, and FOXP3+/CD4+ infiltrate ratios in various tumor subtypes; however, considerable heterogeneity was observed even amongst tumors of the same histologic subtype [113]. Concordantly, microarray analysis of an MPNST-derived cell line versus normal human SCs demonstrated downregulation of genes related to MHC expression and presentation [122], which could account for the absence of T-cell infiltration in some MPNST.

The molecular mechanisms underlying MHC downregulation in MPNST remain unclear, but data suggest that the transcriptional modulation of co-activators and chaperone proteins that regulate MHC expression may be involved [123]. A recent study showed that PRC2 inactivation, a frequent genetic event in MPNST, resulted in an immune-excluded microenvironment and ICB resistance by reprograming the chromatin landscape, disrupting chemokine production, and impairing antigen presentation and T-cell priming [124]. Consistent with these findings, whole genome sequencing, coupled with transcriptomic and methylation profiling of 95 NF1-related tumors, showed a significant association between H3K27 trimethylation (H3K27me3) status and immunophenotype [23]. H3K27me3 loss was strongly correlated with decreased infiltration of immune cells into the TME, downregulation of granzyme expression, and decreased activation of adaptive immunity, while H3K27me3 retention was associated with an immune-cell rich phenotype. Furthermore, loss of H3K27me3 immunoreactivity has been found to be associated with inferior overall survival, indicating the possible utility of H3K27 trimethylation as a prognostic biomarker in MPNST [125].

Spatial gene expression profiling of human PNSTs across the neurofibroma to MPNST continuum revealed that ANNUBPs exhibited enhanced signatures of antigen presentation and T-cell infiltration, while the TME in MPNST becomes immune-excluded with an increased expression of genes associated with immune exhaustion [115]. Notably, neurofibromas contiguous with MPNST were found to harbor distinct gene expression profiles characterized by signatures of impaired antigen presentation, which if validated prospectively, may have utility as potential biomarkers to identify neurofibroma precursors at high risk of undergoing malignant transformation [115]. Further elucidation of the molecular mechanisms governing T-cell recruitment and function within the PNST microenvironment is essential, as this could reveal novel avenues for diagnosis, risk-adapted clinical management, and therapy.

Mast cells are strongly enriched in MPNST and other neural crest-derived malignancies, including melanoma [111,126]. Additionally, NF1-associated MPNST are enriched for c-kit, a marker of mast cells, compared to sporadic MPNST (Figure 2B). However, studies investigating the prognostic significance of mast cell density in MPNST showed no correlation between mast cell infiltration and overall survival [127]. Further studies are needed to establish the functional consequences of mast cell infiltration and/or depletion in preclinical MPNST GEMMs.

Macrophages are one of the most abundant immune cell types in the MPNST microenvironment [57] (Figure 2B). In multiple human sarcomas, including MPNST, M2 macrophages (anti-inflammatory/pro-tumorigenic) outnumber M1 macrophages (pro-inflammatory/anti-tumorigenic) by a mean ratio of 6:1 [128]. Notably, this contrasts with the M1-dominated population of macrophages observed in PNF [101]. A preclinical study of PLX3397, a selective c-Fms and c-kit inhibitor, combined with rapamycin, a TORC1 inhibitor, to deplete tumor-promoting macrophages, showed efficacy with an overall reduction in MPNST cell proliferation [129]. Furthermore, a multicenter phase I of pexidartinib, a CSF-1 receptor inhibitor targeting the polarization of tumor-associated macrophages in MPNST, combined with sirolimus, an mTOR inhibitor, showed safety and overall clinical benefit in 12 of 18 subjects [130,131], and a subsequent phase II study is currently underway (NCT02584647). Collectively, these findings suggest a pro-tumorigenic role of macrophages in MPNST and provide a novel therapeutic strategy for MPNST treatment.

## 7. Conclusions and Future Directions

The PNST microenvironment is shaped by dynamic interactions between *Nf1-/-* SCs and *Nf1+/-* immune cells, which modulate both the initiation and progression of NF1-associated tumors. In this review, we have summarized the current state of the field and highlighted key unanswered questions surrounding the role of *Nf1* gene dose on immune cell function, as well as the putative impact of SC lineage states on immune cell recruitment and function along the neurofibroma to MPNST continuum. We have also discussed emerging evidence that challenges the conventional paradigm of a strictly pro-tumorigenic role for germline *NF1* mutations, suggesting instead a more nuanced role of *NF1* haploinsufficiency within the tumor field: on one hand promoting PNF initiation, while on the other hand, also restraining malignant outgrowth.

Collectively, these findings have potential implications for the diagnosis, risk assessment, and treatment of PNSTs. However, much work is still needed to elucidate the temporal roles of immune cell subsets at discrete stages of tumor initiation, progression, and malignant transformation. In particular, the molecular mechanisms mediating T cell trafficking, activation, exhaustion, and exclusion within PNF, ANNUBP, and MPNST remain poorly understood. Continued innovation in the field of single cell and spatial biology will allow such unresolved questions to be addressed with ever increasing resolution, and will undoubtedly facilitate the discovery and clinical translation of novel diagnostic and therapeutic approaches. Preliminary evidence from case reports and early phase clinical trials indicates that immunotherapy may be effective in a subset of NF1-associated MPNST, but robust biomarkers are needed to identify patients most likely to respond to this treatment. Moreover, the potential utility of immunotherapy as a chemopreventative strategy for MPNST remains uncharted, even in preclinical models. However, improved risk stratification and early detection of neurofibromas at high risk of undergoing malignant transformation will be critical to informing future clinical trials with preventative endpoints. Furthermore, a deeper understanding of the cellular and molecular impact of immunomodulatory therapeutics on the PNST microenvironment will be essential for developing new and effective approaches to improve survival and quality of life for persons with NF1.
